# Exploratory investigation of PSCA-protein expression in primary breast cancer patients reveals a link to HER2/neu overexpression

**DOI:** 10.18632/oncotarget.17523

**Published:** 2017-04-29

**Authors:** Theresa Link, Friederike Kuithan, Armin Ehninger, Jan Dominik Kuhlmann, Michael Kramer, Andreas Werner, Axel Gatzweiler, Barbara Richter, Gerhard Ehninger, Gustavo Baretton, Michael Bachmann, Pauline Wimberger, Katrin Friedrich

**Affiliations:** ^1^ Department of Gynecology and Obstetrics, Medical Faculty and University Hospital Carl Gustav Carus, Technische Universität Dresden, Dresden, Germany; ^2^ National Center for Tumor Diseases (NCT), Partner Site Dresden, Dresden, Germany; ^3^ German Cancer Consortium (DKTK), Dresden and German Cancer Research Center (DKFZ), Heidelberg, Germany; ^4^ Department of Pathology, Medical Faculty and University Hospital Carl Gustav Carus, Technische Universität Dresden, Dresden, Germany; ^5^ Medizinische Klinik und Poliklinik I, Medical Faculty and University Hospital, Technische Universität Dresden, Dresden, Germany; ^6^ GEMoaB Monoclonals GmbH, Dresden, Germany; ^7^ Department of Gynecology, Diakonissenkrankenhaus Dresden, Dresden, Germany; ^8^ Department of Gynecology, St. Joseph Stift, Dresden, Germany; ^9^ Department of Gynecology, Elblandkliniken, Dresden, Germany; ^10^ University Cancer Center (UCC) Carl Gustav Carus, Tumor Immunology, Technische Universität Dresden, Dresden, Germany; ^11^ Helmholtz-Zentrum Dresden-Rossendorf (HZDR), Institute of Radiopharmaceutical Cancer Research, Dresden, Germany

**Keywords:** breast cancer, PSCA, HER2/neu, therapeutic target

## Abstract

**Background:**

Prostate stem cell antigen (PSCA) has been suggested as biomarker and therapeutic target for prostate cancer. Recent advances showed that PSCA is up-regulated in other cancer entities, such as bladder or pancreatic cancer. However, the clinical relevance of PSCA-expression in breast cancer patients has not yet been established and is therefore addressed by the current study.

**Methods:**

PSCA-protein expression was assessed in 405 breast cancer patients, using immunohistochemistry (PSCA antibody MB1) and tissue microarrays.

**Results:**

PSCA-expression was detected in 94/405 patients (23%) and correlated with unfavorable histopathological grade (p=0.011) and increased Ki67 proliferation index (p=0.006). We observed a strong positive correlation between PSCA-protein expression and HER2/neu receptor status (p<0.001). PSCA did not provide prognostic information in the analyzed cohort. Interestingly, the distribution of PSCA-expression among triple negative patients was comparable to the total population.

**Conclusion:**

We identified a subgroup of PSCA-positive breast cancer patients, which could be amenable for a PSCA-targeted therapy. Moreover, given that we found a strong positive correlation between PSCA- and HER/neu expression, targeting PSCA may provide an alternative therapeutic option in case of trastuzumab resistance.

## INTRODUCTION

Breast cancer is the most common cancer in women worldwide [1]. There are different therapeutic options for patients with invasive breast cancer, depending on the presented subtype. Systemic treatment for breast cancer consists of chemotherapy, endocrine or targeted therapy, guided by hormone receptor or HER2/neu status and other clinico-pathological features. For HER2/neu positive disease, targeted therapies are available, including monoclonal antibodies (trastuzumab or pertuzumab), the antibody-drug conjugate trastuzumab-emtansine or the tyrosine kinase inhibitor lapatinib. However, during the course of treatment, a number of patients gain resistance to the current therapy. Therefore, the development of innovative biomarker concepts and additional therapeutic strategies for breast cancer patients is of high clinical importance.

Prostate stem cell antigen (PSCA) is located on chromosome 8q24.2, encodes for a 123 amino acid glycosylphosphatidylinositol (GPI)-anchored cell surface protein and belongs to the Thy-1/Ly-6 family. It was originally defined as an upregulated gene in a human prostate cancer LAPC-4 xenograft model [2, 3]. Therefore, subsequent studies on its potential clinical application primarily focused on prostate cancer. It was shown that PSCA is expressed in 94% of all primary prostate cancers and that its expression correlates with advanced clinical stage, invasion to seminal vesicles, capsular invasion and prostate cancer progression to an androgen independent state [2, 4–6]. Moreover, PSCA mRNA detection in the peripheral blood of prostate cancer patients was shown to be of prognostic relevance [7]. Interestingly, besides its diagnostic potential, PSCA was also evaluated as therapeutic target. In this context, immunotherapies, such as the PSCA-mediated re-direction of T-lymphocytes towards prostate tumor cells or PSCA-mediated vaccination strategies have been proposed [8–10].

PSCA shares 30% homology with the stem cell antigen type 2 (SCA-2), which is an immature lymphocyte cell surface marker. However, considering this rather weak homology to SCA-2, PSCA was inaccurately named, since it is actually neither a marker for a stem cell population, nor is PSCA exclusively expressed in prostatic tissue [3]. The function of PSCA is not yet fully understood. It is believed that this protein is associated with the IFNα/β mediated immune response [11]. Subsequent reports showed that PSCA is likewise up-regulated in other cancer entities, such as gallbladder, urinary bladder cancer, renal cell carcinoma, pancreatic cancer or glioma [12–16], while it is down-regulated in others, such as esophageal and gastric cancers [17–20]. The clinical utility of PSCA as a diagnostic marker or therapeutic target has been demonstrated in prostrate, pancreatic and bladder cancer. Hitherto, PSCA-protein expression was described in only a few normal tissues including prostate epithelium, epithelial layers of the urinary bladder, neuroendocrine cells of the stomach and colon, collecting ducts of the kidney and trophoblasts of the placenta, with conflicting reports about its expression in the normal pancreas [21–23]. PSCA mRNA expression is predominantly found in prostate, placenta, kidney and urogenital tissues [21, 22]. This selective expression in normal tissue makes PSCA a suitable target for immunotherapy.

Currently, for breast cancer patients, there is limited data on PSCA. Some of these investigations have an epidemiological focus and report on genetic variation of the PSCA gene and its relation to breast cancer development. In this context, it was reported, for instance, that PSCA single nucleotide polymorphims (e.g. rs2294008 or rs2978974) are associated with increased risk of developing breast cancer [24]. Moreover, there is some evidence by two preliminary studies suggesting that PSCA-protein might be expressed in at least a subpopulation of breast cancer patients [25, 26]. However one of these studies only focusses on a limited set of patients with micropapillary carcinoma of the breast. The other study investigated PSCA-protein in only 20 fresh frozen breast cancer tissues and suggested that PSCA is rather downregulated in malignant compared to healthy breast tissue. Therefore, considering that clinical relevance of PSCA-protein expression in breast cancer patients is still an open and important question, the objective of the current study was to i) analyze expression pattern and compartmental distribution of the PSCA-protein in normal breast tissue and in breast cancer tissue from a comprehensive cohort of 405 clinically documented breast cancer patients ii) to correlate PSCA-expression to the patient's clinicopathological data and, finally, iii) to investigate prognostic significance of PSCA-expression for breast cancer.

## RESULTS

### Immunohistochemical PSCA-staining in breast cancer tissue and normal breast

**Figure 1:**
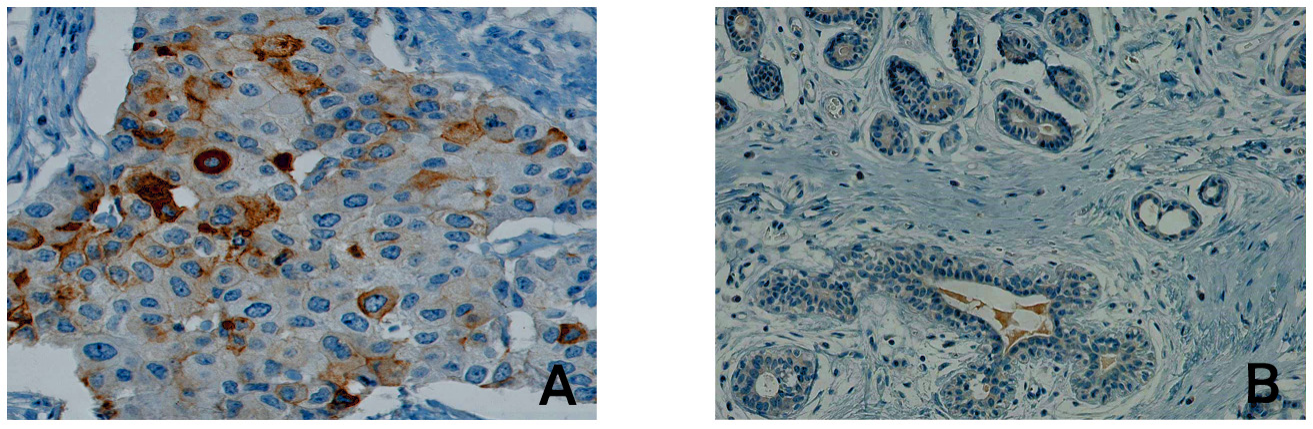
Compartmental distribution of PSCA-expression in breast cancer tissue The Figure shows representative immunohistological PSCA-staining results in (**A**) malignant breast cancer tissue including tumor stroma and (**B**) in non-neoplastic breast tissue. Besides membranous and cytoplasmatic expression of PSCA in invasive breast cancer cells, PSCA-expression is completely absent in stromal cells and in non-neoplastic breast tissue.

**Figure 2:**
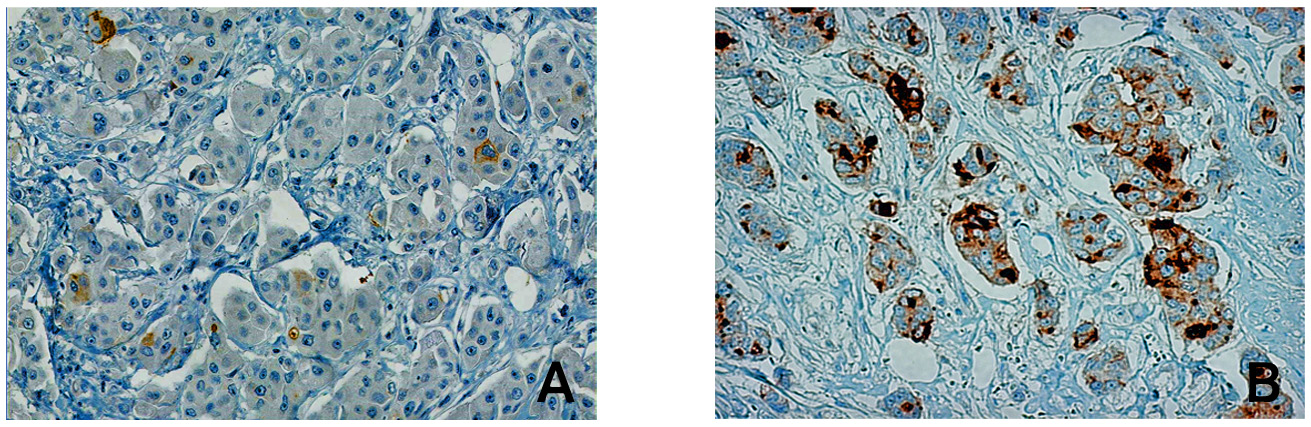
PSCA-expression levels in breast cancer tissue The Figure shows a representative immunohistological PSCA-staining in malignant breast cancer tissue. Among the studied breast cancer patients, PSCA-expression was detected (**A**) in only a few disseminated tumor cells with weak intensity, referred to as “low expression” or (**B**) in a high number of invasive tumor cells, referred to as “high expression”.

In order to exclude a heterogeneous PSCA-expression as well as to assess expression pattern and compartmental distribution of PSCA-protein in breast cancer patients, we performed immunohistochemical PSCA-staining in conventional tissue sections from primary breast tumors. PSCA-staining, which was positive in 4 of 18 samples (22%), appeared homogeneously and was observed with membranous and occasionally cytoplasmic PSCA-reactivity (Figure 1A). PSCA-staining was virtually absent in the vasculature (comprising endothelial cells and smooth muscle cells of the vascular wall), in lymph endothelial cells and in the tumor stroma (comprising fibroblasts, inflammatory cells and fibro-muscular cells, Figure 1B, Figure 2A and 2B). Healthy breast tissue, adjacent to the analyzed tumors (n=18), or breast tissue in specimens from breast reduction surgery (n=2), showed no PSCA-expression (Figure 1B).

### PSCA-expression in breast cancer and its correlation with clinico-pathological features

**Figure 3:**
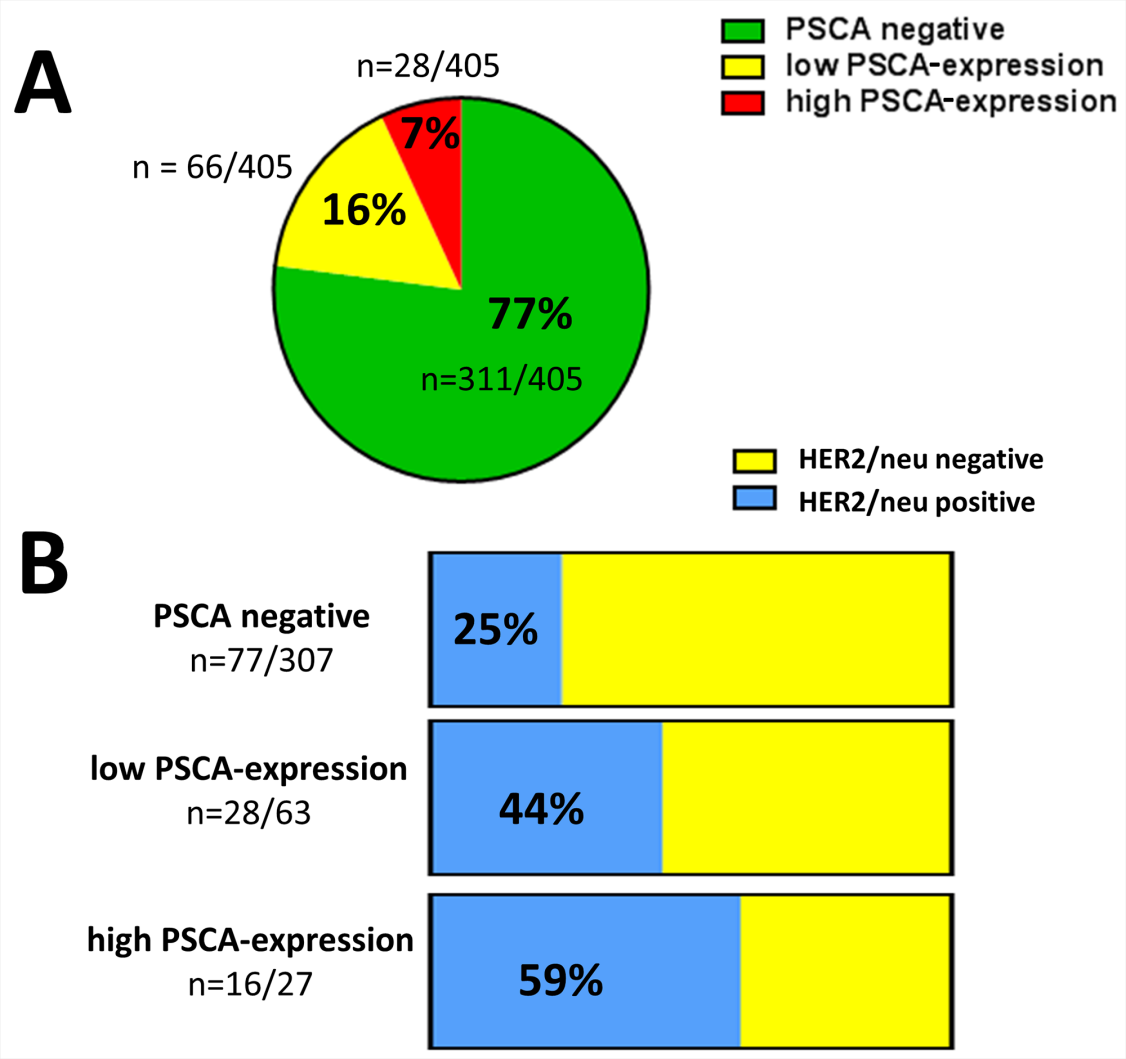
PSCA-expression in breast cancer and its association with HER2/neu status (**A**) The pie chart shows the proportion of low and high PSCA-expression among the studied breast cancer patients according to TMA-analysis. (**B**) The stacked columns show the relative distribution of HER2/neu positive and HER2/neu negative patients with regard to the PSCA-status. Absolute patient numbers of each subgroup are indicated.

Given the spatially homogeneous staining in PSCA-positive breast cancer tissues, we subsequently performed PSCA-tissue microarray analysis in a comprehensive set of clinically documented breast cancer patients (n=405). PSCA-expression was observed with a positivity rate of 23% (94/405 patients). Low PSCA-expression was detected in 16% (66/405 patients) and a high expression was observed in 7% (28/405 patients, Figure 2 and 3A). Subsequently, we correlated PSCA-expression with the patients’ clinico-pathological data (Table 1). PSCA-protein expression was significantly associated with unfavorable histopathological grade (p=0.011) and higher Ki67 proliferation index (p=0.006). No association with age, histological subtype, tumor stage, nodal status, lymphatic-invasion or angio-invasion was observed.

**Table 1:** Correlation of PSCA-expression with clinico-pathological parameter

	PSCA negative	PSCA low	PSCA high	p-value
Her-2/neu status				
HER2-positive	77 (25.1)	28 (44.4)	16 (59.3)	< 0.001
HER2-negative	230 (74.9)	35 (55.6)	11 (40.7)	
histopathological grade				
G1	41 (13.2)	5 (7.6)		0.011
G2	124 (39.9)	25 (37.9)	10 (35.7)	
G3	146 (46.9)	36 (54.5)	18 (64.3)	
Ki67 proliferation index				
0-9%	126 (41.3)	21 (31.8)	4 (14.3)	0.006
10-20%	81 (26.6)	18 (27.3)	11 (39.3)	
>20%	98 (32.1)	27 (40.9)	13 (46.4)	
Age, n (%)				
≤ 50 years	89 (28.6)	11 (16.7)	6 (21.4)	0.112
>50 years	222 (71.4)	55 (83.3)	22 (78.6)	
ER Hormone receptor status				
ER negative	115 (37)	22 (33.3)	16 (57.1)	0.078
ER positive	196 (63)	44 (66.7)	12 (42.9)	
PR Hormone receptor status				
PR negative	131 (42.1)	23 (34.8)	15 (53.6)	0.232
PR positive	180 (57.9)	43 (65.2)	13 (46.4)	
Combination of hormone receptor and HER-2/neu status				
triple negative	75 (24.1)	8 (12.3)	7 (25)	0.108
no triple negative	236 (75.9)	57 (87.7)	21 (75)	
Histological type				
no special type	261 (83.9)	61 (92.4)	25 (89.3)	0.172
invasive lobular	25 (8)	2 (3)		
other types	25 (8)	3 (4.5)	3 (10.7)	
pT-stage				
pT1	130 (41.8)	31 (47)	13 (46.4)	0.690
pT2/pT3/pT4	181 (58.2)	35 (53)	15 (53.6)	
nodal status				
pN0	161 (56.1)	28 (45.2)	12 (46.2)	0.215
pN1 - pN3	126 (40.5)	34 (51.5)	14 (50)	

To sum up, PSCA-expression in breast cancer is associated with unfavorable histopathological grade and increased proliferative activity, however, it does not correlate with other clinico-pathological features.

### PSCA-expression and ER, PR, HER2/neu receptor status

We inquired, whether there is an association between PSCA-expression and receptor status in breast cancer patients. No correlation between estrogen receptor status and progesterone receptor status with PSCA-protein expression was observed. Interestingly, there was a very strong correlation between PSCA-protein expression and HER2/neu receptor status (p<0.001, Table 1). Therefore, in the subgroup of patients with low PSCA-expression, we observed a considerably higher proportion of HER2/neu positivity than in patients with non-detectable PSCA (44% vs. 25%). Interestingly, for patients with high PSCA-expression, the proportion of HER2/neu positivity further increased up to 59% (Figure 3B). Of note, the distribution of PSCA-expression among triple negative patients was comparable to the total population.

Taken together, we demonstrate that PSCA-positive breast cancer patients, especially those with high PSCA-expression, are more likely to overexpress HER2/neu.

### Prognostic significance of PSCA-expression in breast cancer

**Figure 4:**
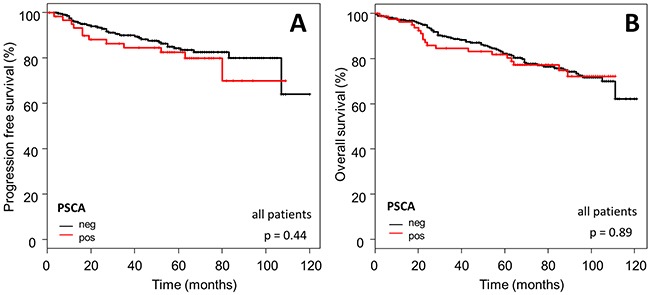
Prognostic relevance of PSCA-expression in the total breast cancer population The Kaplan-Meier plots show (**A**) progression-free survival and (**B**) overall survival of the total study population with PSCA-positive (red curves) vs. PSCA-negative tumors (black curves).

In order to assess prognostic significance of PSCA in breast cancer patients, we correlated PSCA-expression status with the patient's survival data. No association was observed between PSCA-protein expression and PFS (p=0.44) or OS (p=0.89; Figure 4).

**Figure 5:**
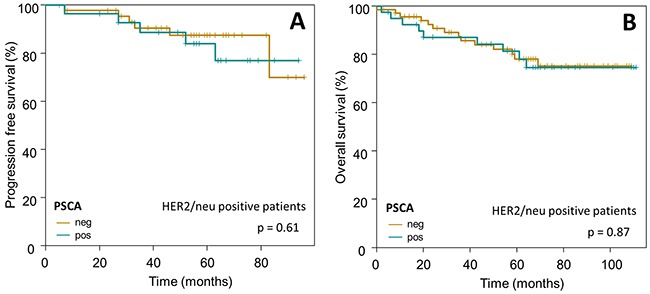
Prognostic relevance of PSCA-expression in HER2/neu positive breast cancer patients The Kaplan-Meier plots show (**A**) progression-free survival and (**B**) overall survival of HER2/neu positive breast cancer patients with PSCA-positive (dark-green curves) vs. PSCA-negative tumors (olive-green curves).

**Figure 6:**
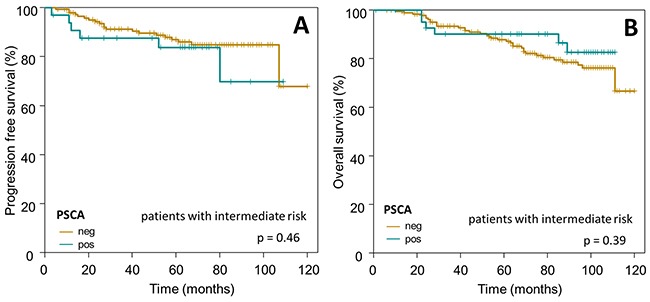
Prognostic relevance of PSCA-expression in breast cancer patients with intermediate risk The Kaplan-Meier plots show (**A**) progression-free survival and (**B**) overall survival of intermediate risk breast cancer patients with PSCA-positive (dark-green curves) vs. PSCA-negative tumors (olive-green curves).

Subsequently, we confined our survival analysis, with additional stratification into clinically relevant subgroups of breast cancer. In accordance to the overall Kaplan-Meier analysis described above, PSCA-protein did not provide prognostic information in the subgroup of i) HER2/neu overexpressing patients (n=110 for OS, p=0.87; n=74 for PFS, p=0.61) or ii) patients with intermediate risk, according to the criteria of St. Gallen 2007 [27] (n=236 for OS, p=0.39; n=182 for PFS, p=0.46, Figure 5 and 6).

In summary, PSCA does not provide prognostic significance in our breast cancer cohort, neither in the overall study population nor in clinically relevant breast cancer subgroups.

## DISCUSSION

In the present study, we demonstrate clinical relevance of PSCA-expression in breast cancer patients. We observed homogenous distribution of PSCA-expressing tumor cells in breast cancer tissue and expected that a TMA-analysis of PSCA-expression will allow conclusions on PSCA-expression across a large number of patients. This assumption was corroborated by the fact that PSCA-positivity rate in conventional breast cancer tissue sections almost exactly corresponded to our TMA-analysis (22% vs. 23%). Although this positivity rate is smaller than in other cancer entities, such as prostate or pancreatic cancer, in which PSCA is up-regulated in 60- 80% [4, 13], we were able to define a subgroup of PSCA-positive breast cancer patients, potentially considered for a PSCA-directed therapy. We observed mostly membranous PSCA-staining of breast cancer cells, which is in accordance with the fact that PSCA is a cell surface antigen [2, 3]. Virtual absence of PSCA-immunoreactivity in the tumor stroma is an interesting finding, since e.g. in prostate cancer, PSCA-protein expression occurs not only in epithelial but also in stromal compartments [28]. Moreover, outside the prostate, immunohistochemical studies in normal human tissues reported on PSCA-staining in the bladder, stomach, colon and kidney [4]. We did not detect PSCA-expression in healthy breast tissues, which is in contrast to a recent study on breast cancer, reporting that PSCA is strongly expressed in normal adjacent tissue but not in the tumor [26]. However, these findings are not directly comparable to our results, since this study included only a very limited number of fresh frozen breast cancer tissues (n=20) and a different staining protocol was applied. Our results indicate that PSCA-expression in breast cancer is restricted to tumor cells and is rather induced as a “neo-antigen” or at least up-regulated in a subgroup of invasive breast cancers.

We did not find an association of PSCA-expression and the patients’ survival, not even in consideration of clinically relevant breast cancer subgroups. This is in accordance to the aforementioned study on breast cancer, which also failed to show any statistically significant associations between PSCA mRNA expression and the patient's clinico-pathological data [26] and could be explained by the heterogeneity of breast cancer disease. Therefore, we conclude that PSCA is not directly suitable as a prognostic biomarker for breast cancer.

Little is known about the function of PSCA. PSCA could possibly interact with other membranous proteins in order to activate downstream signalling pathways. Alternatively, PSCA could be shed from the cellular membrane through the cleavage of its GPI-anchor by phospholipase C and may act as a secreted ligand, which activates other receptor-mediated pathways [29]. Recent functional studies suggested that PSCA could suppress tumor growth and metastasis by inhibiting epithelial-mesenchymal transition (EMT) or by immunomodulation [30–32]. However, since context dependent tumor-suppressive as well as oncogenic functions have been reported, the exact role of PSCA in tumorigenesis is not fully understood. This is in contrast to a similar protein, prostate specific membrane antigen (PSMA), which is highly expressed in the prostate and which has well characterized enzymatic functions (so called “*NAALADase” and folate hydrolase activity)*. PSMA is not only strongly expressed in prostate cancer, it is also upregulated in the neovasculature of solid tumors [33]. Nevertheless, our observations corroborate the assumption, that PSCA-expression parallels a more aggressive and proliferative tumor phenotype and may therefore be rather of oncogenic than tumor-suppressive character in breast cancer. We observed a very strong correlation between PSCA-positivity and HER2/neu overexpression. HER2/neu is a 185-kDa transmembrane oncoprotein, which is overexpressed in about 15-20% of breast cancer patients, who benefit from a HER2/neu directed therapy [34–37]. This finding is of significant clinical interest, since we showed that PSCA-expression status parallels a clinically relevant subgroup of breast cancer patients, eligible for HER2/neu directed therapy. This finding encourages further investigation, to determine whether PSCA-expression could be a response predictor for HER2/neu directed therapy. However, this question could not be addressed in the present study, because a part of our patients were diagnosed before trastuzumab administration became clinical standard for primary breast cancer [36]. Thus, the number of patients, which were HER2/neu overexpressing and actually treated with trastuzumab in our cohort, was too small for reasonable comparison between PSCA-status in trastuzumab responders and non-responders in a statistically substantiated manner.

PSCA has attracted some attention as a therapeutic target. Early clinical trials using full-length antibodies against PSCA in prostate and pancreatic cancer have failed to show signs of efficacy [38, 39]. Consequently, more potent targeting approaches have been developed in a preclinical setting, such as bispecific antibodies for re-direction of T-lymphocytes towards PSCA-positive tumor cells [8, 9, 40, 41]] or chimeric antigen receptor (CAR)-based strategies [42–47]. Of note, a first anti-PSCA CAR trial in pancreatic carcinoma has recently been initiated (NCT02744287). Moreover, radiolabeled antibody derivatives for tumor targeting [48] and PSCA-mediated vaccination strategies have been proposed [49]. Meantime, a variety of approved HER2/neu directed therapies are available, however patients often develop resistance to this kind of treatment [50]. The strong correlation between HER2/neu and PSCA-expression points towards the possibility of targeting PSCA in case of resistance against HER2/neu directed therapy.

Conclusively, we provide the first comprehensive analysis of PSCA-expression and its clinical relevance in breast cancer. Our results suggest that PSCA-based immunotherapeutic and theragnostic approaches may become valuable additions to breast cancer therapy, especially for patients resistant to therapies targeting HER2/neu and to the subset of triple negative patients with PSCA-positive tumors.

## MATERIALS AND METHODS

### Patient characteristics

We analyzed formalin fixed paraffin embedded (FFPE) cancer tissue of 405 primary breast cancers patients, treated according to uniform guidelines in the Regional Breast Cancer Center Dresden between 2003 and 2011. This study was approved by the ethics committee of the University Hospital of Dresden, Germany (EK59032007) and was performed, according to the Declaration of Helsinki. Since this study was performed retrospectively on long term archived FFPE breast cancer tissue without interventions, no patient's consent was required by the guidelines of ethics committee. From each breast cancer, at least two cores were included in the tissue microarrays, resulting in a total number of five microarrays. In order to increase statistical power of comparison of breast cancers with different biological features, two of the tissue microarrays were enriched with triple negative and HER2/neu positive breast cancers cases. In order to exclude heterogeneous expression as well as to assess expression pattern and compartmental distribution of PSCA-expression in breast cancer patients, we performed immunohistochemical PSCA-staining in conventional tissue sections from primary breast tumors (n=18). To check the PSCA-expression in normal breast tissue, the tumor free cancer surrounding tissue in the conventional sections and two breast reduction specimens were analyzed. Table 2 summarizes the patient's clinico-pathological characteristics. Follow up data were available for 366 patients. The follow up time ranged from 1 to 121 months (median follow up 84 months). Twenty four patients died of breast cancer between 10 and 105 month after first surgery (5-year overall survival 82%, 95%-CI [78% - 87%]).

**Table 2:** Patient characteristics at primary diagnosis of breast cancer

	n (%)
Histological subtype	
no special type	347 (85.7)
invasive lobular	27 (6.7)
other subtypes	31 (7.7)
pT-stage	
pT1	174 (43)
pT2	181 (44.7)
pT3	24 (5.9)
pT4	26 (6.4)
nodal status	
pN0	201 (49.6)
pN1 - pN3	174 (43)
unknown	30 (7.4)
histopathological grade	
G1	46 (11.4)
G2	159 (39.3)
G3	200 (49.4)
hormone receptor status	
ER positive	252 (62.2)
ER negative	153 (37.8)
PR positive	236 (58.3)
PR negative	169 (41.7)
Her-2/neu status	
HER2-positive	121 (29.9)
HER2-negative	276 (68.1)
HER2/neu stage unknown	8 (2)
triple negative	90 (22.2)
PSCA-expression	
negative	311 (76.8)
low	66 (16.3)
high	28 (6.9)

### Immunohistochemistry

The expression of PSCA, estrogen and progesterone receptor, HER2/neu and Ki67 was analysed by immunohistochemistry. All staining results were scored semi-quantitatively considering the percentage of positive cells and the staining intensity. The staining result for PSCA was subdivided in negative (no positive cells), low expression (1 to 9% of tumor cells positive) and high expression (>9% positive tumor cells). Table 3 gives an overview of the immunohistochemical analyses, including the antibodies used and the stratification for the staining results. For a more detailed analysis of the outcome, the cases were stratified in three prognosis groups, according to the criteria of St. Gallen 2007 [27] and HER2/neu expression.

**Table 3:** Used antibodies and positivity thresholds for immunohistochemistry

Antigen	Antibody	Dilution	Stratification of staining results
PSCA	MB1[4]	5 ng/μl	1-9% weak positiv; >9% strong positiv
Ki-67	MIB-1 (DAKO)	1:50	0-9%, 10-20%, >20%
estrogen receptor	SP1 (Ventana)	prediluted by manufacturer	threshold for positivity: >1%
progesterone receptor	1E2 (Ventana)	prediluted by manufacturer	threshold for positivity: >1%
HER-2/neu	A0485 (DAKO)	1:800	negative: IRS 0, 1+, 2+ without Her-2/neugene amplificationpositive: IRS 2+ with Her-2/neu geneamplification, IRS 3+; [51]

### Statistical analysis

Statistical analysis was performed by Chi-squared test for comparing the categorical clinico-pathological markers and PSCA-expression. The Cochrane-Armitage-trend test was performed for comparing ordered categorical variables and Kaplan-Meier estimation (log rank test) was used for survival analyses. Overall survival (OS) was defined from day of surgery until death. Progression-free survival (PFS) was defined from day of surgery until disease progression. OS and PFS for patients who did not die (for OS), respective suffer from progression/death (for PFS) during the observational period, were censored at the end of follow-up. Median follow up time was estimated using the reverse Kaplan-Meier-method. The significance threshold was p<0.05 for all tests. Because of the exploratory character of the analyses, no adjustment for multiple testing was performed.

### Ethical approval and consent to participate

This study was approved by the ethics committee of the University Hospital of Dresden, Germany (EK59032007) and was performed, according to the Declaration of Helsinki. Since this study was performed retrospectively on long term archived FFPE breast cancer tissue, no patient's consent was required.

## Abbreviations

CAR: chimeric antigen receptor
EMT: epithelial-to-mesenchymal-transition
FFPE: formalin fixed paraffin embedded
GPI: glycosylphosphatidylinositol
OS: overall survival
PFS: progression free survival
PSCA: prostate stem cell antigen
SCA-2: stem cell antigen type 2 PSMA: Prostate-Specific Membrane Antigen
